# Individual and organizational predictors of health care aide job satisfaction in long term care

**DOI:** 10.1186/s12913-016-1815-6

**Published:** 2016-10-13

**Authors:** Stephanie A. Chamberlain, Matthias Hoben, Janet E. Squires, Carole A. Estabrooks

**Affiliations:** 1 Faculty of Nursing, University of Alberta, 11405-87 Ave, Edmonton, AB T6G 1C9 Canada; 2 University of Ottawa, 451 Smyth Road, Ottawa, ON K1H 8M5 Canada; 3 Ottawa Hospital Research Institute, Centre for Practice-Changing Research (CPCR), 501 Smyth Road, Room 1282, Box 711, Ottawa, ON K1H 8L6 Canada

**Keywords:** Job satisfaction, Care aides, Long term care, Organizational context

## Abstract

**Background:**

Unregulated health care aides provide the majority of direct health care to residents in long term care homes. Lower job satisfaction as reported by care aides is associated with increased turnover of staff. Turnover leads to inferior job performance and negatively impacts quality of care for residents. This study aimed to determine the individual and organizational variables associated with job satisfaction in care aides.

**Methods:**

We surveyed a sample of 1224 care aides from 30 long term care homes in three Western Canadian provinces. The care aides reported their job satisfaction and their perception of the work environment. We used a hierarchical, mixed-effects ordered logistic regression to model the relative odds of care aide job satisfaction for individual, care unit, and facility factors.

**Results:**

Care aide exhaustion, professional efficacy, and cynicism were associated with job satisfaction. Factors in the organizational context that are associated with increased care aide job satisfaction include: leadership, culture, social capital, organizational slack—staff, organizational slack—space, and organizational slack—time.

**Conclusions:**

Our findings suggest that organizational factors account for a greater increase in care aide job satisfaction than do individual factors. These features of the work environment are modifiable and predict care aide job satisfaction. Efforts to improve care aide work environment and quality of care should focus on organizational context.

## Background

The number of older adults over 65 is nearly 15 % of the Canadian population [1]. Close to 300,000 older adults currently live in residential long term care, and this number is expected to increase by ten times in the next 30 years with the rise in Alzheimer’s and related dementias [2]. By 2038 over a million people in Canada will have an age-related dementia [2]. The demand for long term care services will only increase unless the current health care system dramatically shifts towards homecare services and unless we find a cure, an effective preventative strategy, or an effective treatment for age-related dementias.

Residential long term care offers 24-h nursing and meal and laundry services to residents, typically older adults, who are unable to care for themselves in the community. Unregulated workers, known as health care aides, care aides or personal support workers, provide nearly 80 % of direct care to residents in long term care [3, 4]. Care aides bear a heavy workload, assisting in activities of daily living such as feeding, bathing, and dressing and supporting social and emotional needs of residents [5]. Although care aides report a strong sense of job professional efficacy, a sense of personal accomplishment and the belief that their work is meaningful, they are at risk for high levels of burnout [3]. This can ultimately lead to them leaving the long term care workforce.

The long term care sector is characterized by high turnover in all levels of staffing, from direct care providers [6, 7] to top management [8]. As the number of persons entering long term care increases, a focus on staff retention is vital because inconsistent and unstable staffing leads to reduced resident satisfaction [9] and poor quality of care [10, 11]. Care provider job satisfaction has frequently been identified as a predictor of intention to leave a job [12, 13].

### Job satisfaction and organizational context

Job satisfaction is a complex blend of an individual’s emotions, values, and evaluation of task performance. Several theorists define job satisfaction as “the pleasurable emotional state resulting from the appraisal of one’s job as achieving or facilitating the achievement of one’s job values” [14]. Articulating your satisfaction with your job requires you to deliberate on both the work environment and how that environment governs your emotions and task performance [15, 16].

Organizational context describes characteristics of the work environment such as availability of information, leadership, and feedback mechanisms. These characteristics are modifiable and are related to quality of care [17]. Features of an organizational context, such as leaderships and perceived support from peers and management [18], are related to care aide job satisfaction and retention [19]. Care aides feel dissatisfied if they perceive that they have insufficient decision-making capacity or feel that their opinions are not valued by their organization [12]. A nationally representative study of care aides in the US concluded that organizational climate, supervisory behaviour, and time available to complete job tasks are related to overall job satisfaction [20]. Almost all research on job satisfaction in long term care has focused on registered nurses [9, 21–25], even though care aides provide most of the direct care to residents.

No large Canadian studies have examined predictors of care aide job satisfaction in residential long term care. A recent systematic review by Squires et al. [41] found only one Canadian study assessing care aide job satisfaction in long term care [26] and found few high quality studies globally that applied multivariate analysis. The lack of data on care aide job satisfaction in long term care suggests a significant gap in our knowledge of the work environment of this growing workforce.

Our research objective was to conduct the first, large scale analysis of care aide job satisfaction in Canada. We investigated the individual and organizational predictors of job satisfaction in care aides working in long term care. We hypothesized that organizational context variables would be significantly associated with care aide job satisfaction.

## Methods

We used data collected from Phase 1 of the Translating Research in Elder Care (TREC) program [27, 28]. The TREC database contains information on 36 long term care facilities (30 urban and six rural) from three provinces in Western Canada: Alberta, Saskatchewan, and Manitoba. Urban TREC facilities were chosen in a stratified random sample (owner-operator, size, region) [27]. Rural facilities were sampled using convenience sampling. The entire TREC sample consists of 1506 care aide surveys from 36 long term care facilities. We did not include any of the care aide surveys from the six rural nursing homes in this study due to their different sampling technique. We took from the remaining sample of 30 urban homes (*n* = 1381), only those surveys with no missing values in the variables of interest, leaving 1224 care aide surveys in the current sample.

Within TREC, organizational context is assessed with the Alberta Context Tool (ACT). The ACT measures health care providers’ perceptions of modifiable dimensions in organizational context [29]. Informed by the PARIHS framework [30], the ACT has eight dimensions measuring 10 concepts: (1) leadership, (2) culture, (3) evaluation, (4) social capital, (5) informal interactions, (6) formal interactions, (7) structural resources, (8) organizational slack—time, (9) organizational slack—staff, and (10) organizational slack—space (Table 1) [31].

**Table 1:** Individual and Unit Level Scales

Scale	Description	Number of Items	Scoring	Cronbach’s Alpha
Unit Level				
ACT-Leadership	The actions of formal leaders in the unit influence change and excellence in practice.	6	Mean of items on five-point Likert scale (strongly disagree to strongly agree)	0.8369
ACT-Culture	The way that “we do things” in our organization and work units.	6	Mean of items on five-point Likert scale (strongly disagree to strongly agree)	0.7707
ACT-Evaluation	The process of using data to assess group/team performances and to achieve outcomes in organizations or units.	6	Mean of items on five-point Likert scale (strongly disagree to strongly agree)	0.7969
ACT-Formal Interaction	Formal exchanges that occur between individuals working within an organization (unit) through scheduled activities that can promote the transfer of knowledge.	4	Five-point Likert frequency scale (never-almost always). Recode to 0 = no interaction, 1 = interaction. Take count of recoded items.	0.4401
ACT-Informal Interaction	Information exchanges that occur between individuals working within a organization (unit) that can promote the transfer of knowledge.	9	Five-point Likert frequency scale (never-almost always). Recode to 0 = no interaction, 1 = interaction. Take count of recoded items.	0.6820
ACT-Social Capital	The stock of active connections among people.	6	Mean of items on five-point Likert scale (strongly disagree to strongly agree)	0.7430
ACT-Structural Resources	The structural and electronic elements of an organization (unit) that facilitate the ability to assess and use knowledge.	7	Five-point Likert frequency scale (never to almost always). Recode items to 0 = no resource, 1 = resource, take count of recoded items.	0.7329
ACT-Organizational Slack-Staff	The cushion of actual or potential resources that allows an organization (unit) to adapt successfully to internal pressures for adjustments or to external pressures for changes.	3	Mean of items on five-point Likert scale (strongly disagree to strongly agree)	0.9291
ACT-Organizational Slack-Space		2	Mean of items on five-point Likert scale (strongly disagree to strongly agree)	0.8085
ACT-Organizational Slack-Time		4	Mean of items on five-point Likert scale (strongly disagree to strongly agree)	0.8586
Individual Level				
MBI-Exhaustion	Job stress that results in psychological exhaustion.	3	Mean of items on seven-point Likert frequency scale (never-daily)	0.7521
MBI-Cynicism		3	Mean of items on seven-point Likert frequency scale (never-daily)	0.6293
MBI-Professional Efficacy		3	Mean of items on seven-point Likert frequency scale (never-daily)	0.5168

The TREC facility and care unit survey collected information on long term care home characteristics such as bed size, ownership, number, and type of care units. Project coordinators completed the TREC unit and facility survey with managers in the long term care homes (directors of care, administrators) [32]. Trained TREC data collectors surveyed care aides using computer-assisted personal interviewing. The survey measured individual care aide demographic information, organizational context, and job satisfaction.

The scores obtained when the ACT was administered to care aides in long term care showed internal consistency reliability (Table 1) [29]. The validity of the ACT was assessed in the long term care setting using confirmatory factor analysis, analysis of variance, and bivariate associations between each concept and research use. Validity test results indicated that individual care aide scores can be validly aggregated at unit and facility level [29].

### Independent variables

We assessed care aide socio-demographic and education information including sex, age, nationality, and completion of formal health care aide training (possessing a care aide certificate). Work-related characteristics included time worked as a care aide (years), time worked in the unit (years), and shift worked most of the time (day, evening, night). We assessed organizational context using the 10 concepts within the ACT (leadership, culture, evaluation, social capital, informal interactions, formal interactions, structural resources, organizational slack—time, organizational slack—staff, organizational slack—space).

We used the Maslach Burnout Inventory (MBI)-General Survey short form to evaluate the risk of burnout in care aides. This version of the MBI consists of nine items divided into three dimensions or subscales, emotional exhaustion, cynicism, and professional efficacy [27]. We calculated the score for each subscale by taking the mean of the three items. We assessed the internal consistency for the MBI subscales in this care aide population using Cronbach’s alpha. The subscale of emotional exhaustion had acceptable internal consistency (α = 0.752), cynicism and professional efficacy had lower internal consistencies (α = 0.629 and 0.517 respectively).

Unit survey variables included the type of unit where the care aide worked: general long term care, secure dementia, mental health, or combined long term care and dementia. Total number of resident beds was used to define the facility-level variable of size: small (35–79 beds), medium (80–120 beds), and large (>120 beds). The facility-level variable of owner-operator model was recorded as private for profit, public not for profit, or voluntary not for profit.

### Dependent variable

In the care aide survey, job satisfaction was measured with a single item. Previous studies support the psychometric properties of a single-item measure for overall job satisfaction [30, 31, 33]. Care aides were asked to rate their overall job satisfaction on a five-point Likert scale from strongly disagree (1 point) to strongly agree (5 points).

### Analysis

We used a hierarchical, mixed-effects ordered logistic regression to model the relative odds of job satisfaction with respect to care aide, unit, and facility characteristics. We retained the ordered nature of the job satisfaction scale (i.e., five response categories) and used a three-level hierarchical analysis to account for the clustering of care aide responses in units, and units in facilities [34]. The model has two random intercepts: the first is the facility and the second is the unit. Our interpretation of the care aide job satisfaction takes into account the ordered nature of the job satisfaction scale. For example, an adjusted odds ratio (AOR) of 1.3 for the leadership score means that one unit increase in leadership score makes it 1.3 times more likely to be in a lower job satisfaction category compared to an unchanged leadership score, if all other variables in the model are held constant.

We based our selection of variables on statistical selection following bivariate analysis and the available literature, most of which suggests that features of the work environment are related to job satisfaction [26, 35–37]. We conducted both bivariate analysis and multivariate analysis of all independent variables and job satisfaction. For the final model, we report the unadjusted and adjusted odds ratios and 95% confidence intervals. The descriptive statistics and logistic regression analysis were performed using Stata version 13.0 (StataCorp LP, College Station, Texas).

## Results

We studied 1224 care aides from 30 urban nursing homes participating in the TREC program (July 2009–June 2010; Table 2).

**Table 2:** Characteristics of Sample

	Overall (1224)	Alberta (757)	Saskatchewan (181)	Manitoba (286)	*P*-value
Dependent Variable					
Job Satisfaction					
Strongly Disagree	13 (1.06)	6 (0.79)	7 (3.87)	0	
Disagree	56 (4.58)	34 (4.49)	9 (4.97)	13 (4.55)	
Neither Agree or Disagree	105 (8.58)	58 (7.66)	28 (15.47)	19 (6.64)	
Agree	685 (55.96)	422 (55.75)	97 (53.59)	166 (58.04)	
Strongly Agree	365 (29.82)	237 (31.31)	40 (22.10)	88 (30.77)	
Gender					
Female	1131 (92.40)	702 (92.73)	176 (97.24)	253 (88.46)	0.002
Male	93 (7.60)	55 (7.27)	5 (2.76)	33 (11.54)	
Age					
< 20	6 (0.49)	4 (0.53)	0	2 (0.70)	0.838
20–29	133 (10.87)	86 (11.36)	24 (13.26)	23 (8.04)	
30–39	266 (21.73)	160 (21.14)	39 (21.55)	67 (23.43)	
40–49	400 (32.68)	246 (32.50)	60 (33.15)	94 (32.87)	
50–59	325 (26.55)	203 (26.82)	43 (23.76)	79 (27.62)	
> 60	94 (7.68)	58 (7.66)	15 (8.29)	21 (7.34)	
Born in Canada					
Yes	487 (39.79)	266 (35.14)	140 (77.35)	81 (28.32)	<0.001
No	737 (60.21)	491 (64.86)	41 (22.65)	205 (71.38)	
English as first language					
Yes	627 (51.23)	363 (47.95)	114 (79.56)	120 (41.96)	<0.001
No	597 (48.77)	394 (52.05)	37 (20.44)	166 (58.04)	
HCA Certificate					
Yes	1036 (84.64)	623 (82.30)	149 (82.32)	264 (92.31)	<0.001
No	188 (15.36)	134 (17.70)	32 (17.68)	22 (7.69)	
Time worked as HCA (mean, SD)	10.941 (8.73)	10.153 (8.66)	12.40 (9.12)	12.10 (8.45)	<0.001
Shift worked most of the time					
Day	590 (48.20)	354 (46.76)	92 (50.83)	144 (50.35)	0.015
Evening	484 (39.54)	322 (42.54)	68 (37.57)	94 (32.87)	
Night	150 (12.25)	81 (10.70)	21 (11.60)	48 (16.78)	
Unit Type					
General LTC	797 (65.11)	408 (53.90)	143 (79.01)	246 (86.01)	<0.001
Secure dementia/Alzheimer’s	349 (28.51)	293 (38.71)	38 (20.99)	18 (6.29)	
Mental health	10 (0.82)	10 (1.32)	0	0	
Combined LTC and dementia	68 (5.56)	46 (6.08)	0	22 (7.69)	
NH Size					
Small (35–79 beds)	233 (19.04)	173 (22.85)	60 (33.15)	0	<0.001
Medium (80–120 beds)	288 (23.53)	78 (10.30)	59 (32.60)	151 (52.80)	
Large (>120 beds)	703 (57.43)	506 (66.84)	62 (34.25)	135 (47.20)	
NH Owner/Operator					
Public	349 (28.51)	349 (46.10)	0	0	<0.001
Private for Profit	267 (21.81)	133 (17.570	48 (26.52)	86 (30.07)	
Voluntary	608 (49.67)	275 (36.33)	133 (73.480	200 (69.93)	

The majority of participants were women, 40–49 years of age, and born outside Canada (Table 2). Care aides surveyed in both the complete sample of urban homes in the TREC program and in this sample report feeling *satisfied* in their job [3]. In our sample, very few (5.64 %) indicated that they are unsatisfied with their job (*disagreed or strongly disagreed* with the statement “In general, I am satisfied with my present job”). Table 2 shows care aide job satisfaction. All variables from Table 2 were included in the multilevel analysis.

The intraclass correlation at the facility (Estimate = 0.019, Standard Error = 0.031) and unit level (Estimate = <0.001, Standard Error = <0.001) demonstrated minimal clustering of care aide responses at either facility or unit level.

### Bivariate analysis

The bivariate analysis showed that 14 of 27 variables were significantly associated with care aide job satisfaction (Table 3). Bivariate analysis of *shift worked most of the time* was not statistically significant and was not retained in further analysis. Care aide characteristics (age, sex) and facility characteristics (owner/operator model, bed size) were not significantly associated with job satisfaction in bivariate analysis. However, they were included as covariates in the final analysis because previous research found significant associations between those variables and care aide job satisfaction [13, 38, 39].

**Table 3:** Individual, Unit, and Facility Characteristics Associated with Care Aide Job Satisfaction

	Adjusted OR		Unadjusted OR	
	OR (95 % CI)	*P*-Value	OR (95 % CI)	*P*-Value
Fixed Effects				
Gender				
Female	1.00 (0.65–1.55)	0.980	0.83 (0.55–1.25)	0.374
Age				
20–29	1.27 (0.25–6.54)	0.776	0.687 (0.15–3.09)	0.624
30–39	1.65 (0.32–8.35)	0.548	0.96 (0.22–4.24)	0.958
40–49	1.67 (0.33–8.42)	0.535	1.15 (0.26–5.06)	0.852
50–59	1.75 (0.35–8.89)	0.498	1.10 (0.25–4.86)	0.896
> 60	1.68 (0.32–8.84)	0.536	1.18 (0.26–5.41)	0.825
Born in Canada				
No	0.91 (0.68–1.22)	0.547	1.94 (1.53–2.45)	<0.001
NH Size				
Medium (80–120 beds)	1.12 (0.75–1.68)	0.584	1.14 (0.76–1.70)	0.515
Large (>120 beds)	0.82 (0.55–1.21)	0.319	1.22 (0.87–1.73)	0.252
NH Owner				
Private for Profit	0.94 (0.66–1.34)	0.735	0.81 (0.57–1.18)	0.289
Voluntary not for profit	0.90 (0.67–1.21)	0.493	0.90 (0.66–1.21)	0.485
Unit Type				
Secure Dementia/Alzheimer’s	0.71 (0.54–0.95)	0.022	1.00 (0.75–1.34)	0.961
Mental Health	0.41 (0.10–1.57)	0.195	0.45 (0.12–1.68)	0.233
Combined LTC and Dementia	0.83 (0.48–1.44)	0.512	0.60 (0.34–1.04)	0.069
ACT Concept				
Leadership	1.30 (1.04–1.64)	0.021	2.95 (2.43–3.58)	<0.001
Culture	2.86 (2.09–3.90)	<0.001	6.30 (5.23–8.40)	<0.001
Evaluation	0.99 (0.80–1.24)	0.981	2.21 (1.84–2.66)	<0.001
Formal Interaction	0.94 (0.76–1.13)	0.513	1.25 (1.08–1.50)	0.003
Informal Interaction	0.96 (0.87–1.04)	0.317	1.13 (1.05–1.22)	0.001
Social Capital	1.50 (1.13–2.00)	0.005	3.92 (3.10–4.96)	<0.001
Structural and Electronic Resources	1.06 (0.99–1.16)	0.099	1.31 (1.22–1.39)	<0.001
Organizational Slack—Staff	1.23 (1.06–1.53)	0.002	1.80 (1.61–2.01)	<0.001
Organizational Slack—Space	1.14 (1.00–1.31)	0.050	1.65 (1.47–1.85)	<0.001
Organizational Slack—Time	1.27 (1.06–1.53)	0.010	2.15 (1.87–2.46)	<0.001
MBI Scales				
MBI-Exhaustion	0.85 (0.77–0.93)	<0.001	0.68 (0.65–0.75)	<0.001
MBI-Cynicism	0.87 (0.80–0.96)	0.005	0.72 (0.67–0.77)	<0.001
MBI-Professional Efficacy	1.33 (1.16–1.53)	<0.001	1.76 (1.55–2.00)	<0.001
	Estimate	SE	95 % CI Lower	95 % CI Lower
Facility	0.0028	0.017	<0.001	677.49
Unit	<0.001	<0.001	-	-

Reference groups are: Gender (Male), NH Size (Small), Unit Type (General LTC), Born in Canada (Yes), NH Owner/Operator (Public)

### Multivariate analysis

At the individual level, higher MBI-Emotional Exhaustion (AOR = 0.85, 95 % CI: 0.77–0.93), and higher MBI-Cynicism (AOR = 0.87, 95 % CI: 0.80–0.96) were associated with lower care aide job satisfaction. Higher MBI-Professional efficacy (AOR = 1.33, 95 % CI: 1.16–1.53) was associated with higher care aide job satisfaction.

At the unit level, six organizational context concepts were associated with care aide job satisfaction: leadership, culture, social capital, organizational slack—staff, organizational slack—space, and organizational slack—time. The ACT concept of organizational culture had the highest odds of higher care aide job satisfaction (AOR = 2.86, 95 % CI: 2.09–3.90), followed by social capital (AOR = 1.50, 95 % CI: 1.13–2.00), and leadership (AOR = 1.30, 95 % CI: 1.04–1.64). Organizational slack—time (AOR = 1.27, 95 % CI: 1.06–1.53), organizational slack—staff (AOR = 1.23, 95 % CI: 1.06–1.53), and organizational slack—space (AOR = 1.14, 95 % CI: 1.00–1.31), also were associated with higher care aide job satisfaction. Care aides working in secure dementia/Alzheimer’s units had lower odds of higher job satisfaction scores (AOR = 0.71, 95 % CI: 0.54–0.95). None of the facility variables, including bed size and owner/operator model, impacted care aide job satisfaction.

## Discussion

Care aide job satisfaction is an important factor in intent to leave the job, staff retention, and quality of care. We found that both individual and unit level predictors were significantly related to care aide job satisfaction. Facility level characteristics did not increase the odds of care aides having higher satisfaction in their current job, nor did care aide demographic characteristics, including age, sex, and nationality. This is consistent with a recent report that suggests that, while both organizational and individual factors are important, organizational elements are more closely related to care aide job satisfaction [40, 41].

Care aides bear enormous responsibility in attending to both physical and emotional needs of residents, and often report feeling rushed when trying to provide the necessary care [42]. We found an inverse relationship between care aide satisfaction and emotional exhaustion, where a higher score on the MBI-emotional exhaustion scale indicated that a care aide felt emotionally overloaded with the work. Although care aides experience worrying rates of burnout [3], they consistently report high job satisfaction [43]. Care aides develop individual coping strategies in the presence of stress and conflict on the job and the capacity to cope in the face of stress can result in improved job satisfaction [44].

An important finding from this study was the effect of organizational context on care aide job satisfaction. A supportive work environment that emphasizes the importance of supervisor leadership and decision-making autonomy for direct care staff increases care aide job satisfaction [18, 37]. Elements of the care unit context (leadership, culture, social capital, organizational slack—staff, organizational slack—time) are related to care aide job satisfaction. Leadership as a significantly associated with care aide job satisfaction is unsurprising, given the research suggesting that support from leaders influences the quality of work environment for staff on the unit [17, 18]. Our finding that organizational slack in staff and time were associated with increased job satisfaction supports repeated calls by front line workers and their managers in the long term care sector for increased staffing to provide optimal quality of care to residents. When care aides feel they do not have to enough time to complete all their tasks, their job satisfaction is reduced [20].

We found that an increase in professional efficacy, the feeling of personal achievement or accomplishment, increased overall job satisfaction. This is consistent with reports of significant associations between work engagement, satisfaction, and professional efficacy [45]. The strengths of emotional exhaustion, cynicism, and professional efficacy as associated with job satisfaction enhance our confidence in the validity of the MBI subscales as predictors for our sample. Future research on the care aide population is necessary to understand the relationship between burnout and job satisfaction, as research also suggests that a lower level of satisfaction is a strong predictor of burnout [46–48].

The significance of the unit level predictors validates continued efforts to examine quality of care at the resident care unit level [32]. We located only a few studies of care aides in long term care that assessed job satisfaction at this unit level. We found that unit level features of the organizational context such as greater leadership, autonomy, and support for shared ideas are significantly associated with increased job satisfaction of care aides in long term care [18]. Our analysis identified modifiable features of the organizational context that can improve care aide job satisfaction. These results add support to the growing body of research which suggests that quality improvement activities should be focused at the unit-level to improve staff and resident outcomes [32, 49–51]. Many of the features of an effective unit (leadership, culture, information, interdependence of care team) are distinct domains captured in our conceptualization of organizational context [29]. Modifiable features of the organization’s context offer targeted areas for quality improvement and performance management. Future efforts to improve care aide job satisfaction can be effectively targeted to the unit level and address the features of the unit context.

Our findings indicate that job satisfaction did not significantly vary across units and facilities. Facility and unit intraclass correlation coefficients (ICCs) were low and not statistically significant. This is surprising, as we know from previous research that ACT concepts significantly cluster within care units and facilities (i.e., the variance of ACT concepts within units and facilities is smaller than the variance of ACT concepts between units and facilities) [32, 52]. However, although the ACT concepts (leadership, culture, social capital) were significantly associated with higher care aide job satisfaction, job satisfaction itself does not show significant cluster-effects within facilities and units. Care aide job satisfaction is an individual phenomenon, rather than a shared one at the unit or facility level. Therefore, we assume that in addition to organizational context characteristics, individual variables not measured within our study (such as compensation, employment benefits, hours of care per patient) may strongly influence care aide’s job satisfaction [34, 36, 53]. For this analysis we did not have available concepts such as organizational citizenship behaviour or work engagement, both of which have been associated with job satisfaction [37, 43, 54]. These concepts are included in the next phase of TREC care aide survey data collection and may better explain how individual variables, context and care aide job satisfaction are associated.

### Study limitations

Strengths of the study include a large sample of 1224 care aides from 30 randomly selected long term care homes. Scores obtained with our measure of organizational context have been shown to be reliable and valid when administered to the care aide population. To our knowledge, this is the first study to specifically examine care aide job satisfaction in long term care using unit level measurement of organizational context features. Limitations of our study analysis include its cross-sectional nature, which limits our ability to assess whether temporal changes in organizational context affect care aide job satisfaction. We analyzed care aides from long term care homes in the Prairie provinces in Western Canada, therefore our results may not be generalizable beyond this study sample. Our measure of care aide burnout (Maslach Burnout Inventory) had two subscales (cynicism, professional efficacy) with Cronbach’s alpha’s that were below the recommended acceptability level of 0.70 in our sample of care aides. This suggests that further psychometric assessment is needed for this measure in the care aide population in long term care. Cronbach’s alpha for the organizational context measures of formal and informal interactions were below the recommended acceptability level and should be interpreted with caution. We used a single item to measure job satisfaction which is sufficient to provide a global report of job satisfaction [30]. A single-item measure of care aide job satisfaction was appropriate for our focus on overall satisfaction, as opposed to specific components of satisfaction [55]. Although single-item measures are psychometrically sound, we cannot assess whether these results would be consistent if a multi-item scale was used.

## Conclusions

Our results suggest that both individual and unit level factors are associated with care aide job satisfaction in long term care. Organizational factors account for a greater increase in care aide job satisfaction than do individual factors. We demonstrated that efforts to improve unit level context features of leadership, culture, social capital, organizational slack—staff, organizational slack—space, and organizational slack—time are likely to improve care aide job satisfaction. Ultimately such efforts could improve the quality of care provided to residents in long term care. These are modifiable features of the organizational context that are present at the unit level and that largely influenced care aide job satisfaction. Future efforts aimed at improving care aide job satisfaction should focus on the unit level and how features of the work environment can improve care aide job satisfaction.

## Abbreviations

ACT: Alberta Context Tool
MBI: Maslach Burnout Inventory
TREC: Translating Research in Elder Care
